# Hairy cell leukemia variant and WHO classification correspondence Re: 5^th^ edition WHO classification haematolymphoid tumors: lymphoid neoplasms

**DOI:** 10.1038/s41375-024-02280-0

**Published:** 2024-06-08

**Authors:** Michael Grever, Leslie Andritsos, Mirela Anghelina, Evgeny Arons, Versha Banerji, Jacqueline Barrientos, Seema A. Bhat, James Blachly, Alessandro Broccoli, Timothy Call, Claire Dearden, Sascha Dietrich, Monica Else, Narendranath Epperla, Andrei Fagarasanu, Brunangelo Falini, Francesco Forconi, Alessandro Gozzetti, Paul Hampel, David J. Hermel, Sunil Iyengar, James B. Johnston, Gunnar Juliusson, Robert J. Kreitman, Francesco Lauria, Gerard Lozanski, Christopher C. Oakes, Sameer A. Parikh, Jae Park, Graeme Quest, Kanti Rai, Farhad Ravandi, Tadeusz Robak, Kerry A. Rogers, Alan Saven, John F. Seymour, Tamar Tadmor, Martin S. Tallman, Constantine S. Tam, Enrico Tiacci, Xavier Troussard, Bernhard Wörmann, Clive S. Zent, Thorsten Zenz, Pier Luigi Zinzani

**Affiliations:** 1 Department of Internal Medicine, Division of Hematology, The Ohio State University, Columbus, OH USA; 2 Division of Hematology Oncology, University of New Mexico Comprehensive Cancer Center, Albuquerque, NM USA; 3 Center for Biostatistics, The Ohio State University, Columbus, OH USA; 4 Laboratory of Molecular Biology, National Cancer Institute, NIH, Bethesda, MD USA; 5 Department of Internal Medicine, Max Rady College of Medicine, Rady Faculty of Health Sciences, University of Manitoba, and Paul Albrechtsen Research Institute CancerCare Manitoba, Winnipeg, MB Canada; 6 Mount Sinai Medical Center Miami, Miami, FL USA; 7 IRCCS Azienda Ospedaliero-Universitaria di Bologna, Policlinico “Sant’Orsola-Malpighi”, and Department of Medical and Surgical Sciences - University of Bologna, Bologna, Italy; 8 Division of Hematology, Mayo Clinic, Rochester, MN USA; 9 The Royal Marsden NHS Foundation Trust, London, UK; 10 University Hospital of Düsseldorf, Düsseldorf, Germany; 11 The Institute of Cancer Research, London, UK; 12 University of Alberta, Edmonton, AB Canada; 13 Institute of Hematology and Center for Hemato-Oncological Research (CREO), University of Perugia and Santa Maria della Misericordia Hospital, Perugia, Italy; 14 Cancer Sciences and Haematology Department, University of Southampton and Cancer Care, Haematology Department, Southampton NHS University Hospital Trust, Southampton, UK; 15 Department of Medicine, Surgery and Neurosciences, University of Siena Policlinico S Maria alle Scotte, Siena, Italy; 16 Division of Hematology and Oncology, Scripps Clinic, La Jolla, CA USA; 17 Department of Medicine, University of Manitoba, Winnipeg, MB Canada; 18 Lund University/Dept of Hematology, Skåne University Hospital, Lund, Sweden; 19 University of Siena, Siena, Italy; 20 Department of Pathology, The Ohio State University Medical Center, Columbus, OH USA; 21 Memorial Sloan Kettering Cancer Center, New York City, NY USA; 22 Pathology and Molecular Medicine, Queen’s University and Kingston Health Sciences Centre, Kingston, ON Canada; 23 Zucker School of Medicine at Hofstra/Northwell, New Hyde Park, NY USA; 24 Department of Leukemia, University of Texas MD Anderson Cancer Center, Houston, TX USA; 25 Department of Hematology, Medical University of Lodz, Copernicus Memorial Hospital, Lodz, Poland; 26 Peter MacCallum Cancer Centre, Royal Melbourne Hospital & University of Melbourne, Melbourne, VIC Australia; 27 Hematology Unit, Bnai Zion Medical Center, and the Ruth and Bruce Rappaport Faculty of Medicine, Technion, Haifa, Israel; 28 Robert H. Lurie Comprehensive Cancer Center of Northwestern University, Chicago, IL USA; 29 Alfred Hospital and Monash University, Melbourne, VIC Australia; 30 Department of Hematology, Centre Hospitalier Universitaire Cote de Nacre, Caen, France; 31 The Charité – Universitätsmedizin, Berlin, Germany; 32 James P. Wilmot Cancer Institute and Department of Medicine, University of Rochester Medical Center, Rochester, NY USA; 33 Dept. of Medical Oncology & Haematology, Univerity Hospital and University of Zurich; The LOOP Zurich – Medical Research Center, Zurich, Switzerland

**Keywords:** Hairy cell leukaemia, Diagnosis

## To the Editor:

In 2022, Alaggio and colleagues revised the WHO Classification of Haematolymphoid Tumors resulting in elimination of the provisional diagnostic categories of Hairy Cell Leukemia Variant (HCLv) and B Prolymphocytic Leukemia [1]. A new diagnostic category Splenic B Cell Lymphoma/Leukemia with Prominent Nucleoli (SBL/LPN) was established to include both cases of HCLv and the very rare CD5 (-) B Cell Prolymphocytic Leukemia (B-PLL). While some other cases of splenic lymphoma might also be included, the ultimate goal to delineate biologically-defined entities may improve therapy of these often over-lapping clinical diseases. Coupland and colleagues recently provided a comprehensive assessment of the potential therapeutic opportunities that might emerge from reorganizing the classification of these clinical entities to a system incorporating evidence-based disease information [2]. As an international group of experts in the field of HCL and HCLv, we are concerned that this reassignment of patients with HCLv to a category including other rare B cell malignancies may impair the identification of new specific targeted therapeutic strategies that are urgently needed. While HCLv is not a homogenous clinical entity and lacks a single pathognomic molecular test, subsets of this disease have specific molecular targets that can serve as potential therapeutic opportunities for improved outcome. Discerning the oncogenetic mechanisms and targets will necessitate extensive collaboration and work between clinicians and basic scientists to optimize the therapeutic benefit from a revised classification.

Tremendous progress has been made in the treatment of classic hairy cell leukemia (HCLc) over the past six decades [3]. The introduction of purine nucleoside analogs and newer targeted agents improved survival in HCLc from slightly more than 4 years following diagnosis to projected survival close to normal life span. In contrast, patients with the distinct rare entity previously called “hairy cell leukemia variant (HCLv)” have a more aggressive clinical course with poorer response to therapies. This entity was initially described by Cawley in 1980, and subsequently major biological and clinical differences were identified between the classic and the variant forms of this disease. Patients with HCLc present with pancytopenia, splenomegaly, monocytopenia, and a marked increased risk for serious infection. In contrast, patients with HCLv present with splenomegaly, elevated peripheral leukemic cells with nucleoli, and no monocytopenia [3, 4]. Both entities have an unexplained male predominance with HCLc being more common than the rare HCLv. There are both distinguishing immunophenotypic and immunocytochemical (e.g., Annexin A1 is expressed in HCLc, but not in HCLv) profiles that differentiate these two clinical entities.

Response to purine analogs is also different between these two conditions. HCLc patients initially treated with a purine nucleoside analog show excellent response [3]. Patients with HCLv treated with purine analog alone are much less responsive. Durable remissions require the combination of cladribine plus rituximab [5]. The clinical course of patients with HCLv is more aggressive than HCLc with projected survival of approximately 6–9 years [4, 5]. For patients with HCLc who either relapse or do not respond, the documentation of the *BRAF* V600E mutation affords an opportunity to rescue the patient with a *BRAF* inhibitor plus an anti-CD20 mAb [6]. Unfortunately, the *BRAF* V600E mutation is not found in patients with HCLv [4]. Therefore, this rescue option is not available for patients with the variant if they either fail to respond or relapse.

Extensive investigation of leukemic cells from patients with HCLv have revealed many of the molecular abnormalities that make this less responsive and more aggressive. In HCLv, approximately 30-38% of patients have an abnormal p53 either as a result of deletion or mutation [4, 5]. Additional mutations in leukemic cells show abnormalities in signaling pathways that may afford opportunities for strategic intervention. Mutations in *MAP2K1* found in less than half of the patients enabled the use of a MEK inhibitor to effectively control the aggressive phase of HCLv following failure to respond to multiple agents [7]. A currently open phase 2 trial of the MEK inhibitor binimetinib (NCT04322383) allows HCLv patients to receive an anti-CD20 mAb if needed to eliminate minimal residual disease if and when complete remission is achieved. Exploration of new agents alone and in combination in treating patients with HCLv should be pursued. Introduction of BTK inhibitors has produced responses in patients who have been previously heavily-pretreated with standard agents [8]. Considering the prevalence of p53 abnormalities in patients with HCLv, incorporation of BTK inhibitors may afford an opportunity to achieve a response in resistant disease. Likewise, BCL-2 inhibitors (e.g., venetoclax) may play an increasing role in managing these resistant patients. Early reports indicate that venetoclax has promise as an active agent in relapsed/resistant HCL patients [9, 10]. Determining the optimal agents to use in combination (e.g. which mAb would be more effective) has led to studies incorporating obinutuzumab along with other novel mechanistic anti-leukemic agents.

In the process of exploring novel agents and combinations in treating HCLv, inter-institutional collaboration will be necessary to evaluate new targeted therapy for such rare lymphoma subsets. In the past, a randomized intergroup study evaluated front-line therapy with pentostatin versus alpha-interferon. This study enrolled 356 previously untreated patients in less than 4 years. So, large studies are potentially feasible with inter-institutional collaboration.

International collaboration through the Hairy Cell Leukemia Foundation could also be utilized to recruit a sufficient number of patients to address research questions targeting the development of effective therapeutic regimens. In order to develop targeted therapy for rare clinical entities (e.g., HCLv), it will be important to design studies that enroll well-characterized patients. Therefore, the new classification of patients with HCLv with other B cell lymphoproliferative malignancies may introduce different clinical conditions that may complicate interpretation of efficacy. Elucidation of molecular targets in patient material and the pharmacodynamic impact of treatment will be important in evaluating the efficacy of novel therapy. In order to improve therapy for HCLv in the near future, we will need to track patients with this rare entity and not lose important clinical information as a result of this recent change in the WHO disease classification.
